# Aspartyl Proteinases of Eukaryotic Microbial Pathogens: From Eating to Heating

**DOI:** 10.1371/journal.ppat.1005992

**Published:** 2016-12-22

**Authors:** Antonio Cassone, Anna Vecchiarelli, Bernhard Hube

**Affiliations:** 1 Polo d’innovazione della Genomica, Genetica e Biologia, University of Perugia, Perugia, Italy; 2 Microbiology Section, Department of Experimental Medicine, University of Perugia, Perugia, Italy; 3 Department of Microbial Pathogenicity Mechanisms, Leibniz Institute for Natural Product Research and Infection Biology—Hans Knoell Institute, Jena, Germany; 4 Center for Sepsis Control and Care, University Hospital Jena, Jena, Germany; 5 Friedrich-Schiller-Universität, Jena, Germany; McGill University, CANADA

## The Conserved Structures of Microbial Aspartyl Proteinases

**Fig 1:**
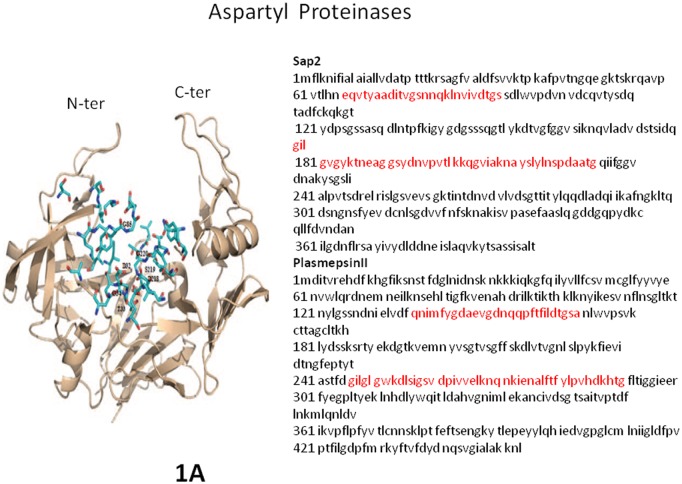
**Left**. The molecular ribbon-like structure of Sap2, a major AP of *Candida albicans*. Note the flexible flaps that control the access to the central region faced and delimited by the two active sites DTGS and DSGT and accommodating an enzyme inhibitor. N-ter is the N-terminus and C-ter the C-terminus of the amino-acid sequence. **Right**. Sequences of Sap2 and plasmepsin II of *Plasmodium falciparum*, which is most similar to Sap2 among the APs of eukaryotic microbial pathogens, showing two regions of high similarity (highlighted in red). The identity of the two whole sequences is 28.2% and their similarity 57.4% (FASTA; MBL Swiss-Prot).

Aspartyl (or aspartic) proteinases (APs) are a class of proteinases (or proteases) highly conserved from retroviruses, including the HIV-1 protease, to mammals, including pepsins, cathepsins, and renins [1]. APs of eukaryotic pathogens usually have two-domain structures, each of which provides a catalytic Asp residue to the active enzymatic site. The N-terminal domain contains a “flap” β-hairpin that overhangs the active site. By virtue of its high flexibility, the flap controls access to the active site [2,3]. AP evolution from a common ancestor is exemplified by similar structures and sequence similarities, with predominantly conserved regions containing the catalytic aspartic residues. Consequently, catalytic mechanisms are similar, indicated by a largely preserved sensitivity to pepstatin A, a prototypal AP inhibitor. Some APs of eukaryotic pathogens are also sensitive to a number of HIV-AP inhibitors effectively used for AIDS therapy [4–6] (Fig 1).

The relevance of APs for the success of eukaryotic pathogens as infectious agents is reflected in the APs’ redundancy and organization in protein families with distinctive but genetically related members [7,8], an evolutionary expansion that appears to have reached a particularly high level of diversification in some fungal organisms [1]. Functionally, this organization enables the pathogen to select the right AP at the right time and in the right place to exploit synergistic effects or to use alternative APs when one is lost or inactive, thereby compensating for the biological cost of having many copies of the same gene [1,7–9].

While the structure of APs is well conserved, their biological functions are extremely broad. Here we highlight two aspects of APs of eukaryotic pathogens: (1) their enzymatic activity (“eating”), which spans from protein degradation as nitrogen source to structural functions or roles in cellular transport, collectively required for growth, cellular functions, fitness and pathogenicity, and (2) their ability to trigger inflammation (“heating”) within the complex of host immune responses and independently on AP enzymatic activity. This last aspect has so far been underappreciated despite the fact that it can also play an important role in pathogenesis and disease control, including vaccination.

Exemplary of such a multifaceted scenario are the APs of *Plasmodium falciparum* (plasmepsins) and *Candida albicans* (secretory aspartyl proteinases), two evolutionarily distant pathogens that, despite their differences, are both characterized by high genetic plasticity and a complex relationship with the human host.

## Plasmepsins

The AP “eating” functions and their consequences for the pathogen–host relationship are well represented by plasmepsins of *P*. *falciparum*. This is a major agent of malaria, a disease with an estimated more than 200 million cases and 438,000 deaths in 2015 [10]. Of the ten *P*. *falciparum* plasmepsins, I–IV are involved in hydrolyzing host hemoglobin and removal of its toxic products, acting together with other non-aspartyl proteinases such as the falcipains and falcilysins within the Haem Degradation Protein (HDP) complex at the intra-erythrocytic stage [8,9]. This process enables the parasite to utilize hemoglobin as an amino acid source and avoid potential damage by iron and haem molecules through the formation of hemozoin crystals. On the other hand, plasmepsin V is involved in the export of malarial effector proteins through the endoplasmic reticulum to the erythrocyte, a crucial activity for protozoan survival [11–12]. In fact, plasmepsin V cleaves a factor named Plasmodial Export Element (PEXEL) that allows for the export of malarial proteins into the host cell. Some of the exported proteins play an important role in virulence and antigen presentation [11–18]. A similar role appears to be played by ASP5, a phylogenetically related AP of *Toxoplasma gondii*, the deletion of which makes *T*. *gondii* unable to cleave the PEXEL-like motif, thereby negatively impacting parasite fitness and virulence in vivo [19–21]. The other five plasmepsins in *P*. *falciparum* are not components of the food vacuole HDP, and their functions are poorly understood. However, plasmepsins VII and X have recently been detected in ookinetes and zygotes of *P*. *falciparum*, and antibodies against these plasmepsins have been shown to reduce the infectivity of *P*. *falciparum* for mosquitoes [22]. For all the above reasons, plasmepsins have become attractive targets for possible chemo- and immuno-therapeutic interventions. In particular, the virulence-attenuated, plasmepsin IV-deleted strain of *P*. *berghei*, an agent of murine malaria, has been suggested as a blood-stage, whole-cell vaccine [23–24]. The plasmepsins’ redundancy and consequent stringent requirements for their use of molecular targets of new plasmodial inhibitors has been critically discussed [9].

## Secretory Aspartyl Proteinases

Several APs are produced, often in a secretory form, by many fungal organisms, including both primary and opportunistic pathogens. For example, a glycosylated AP from *Paracoccidioides brasiliensis*, the agent of a deep-seated mycosis in Latin America, and a non-glycosylated AP from *Coccidioides posadasai*, an agent of coccidioidomycosis in America, have been isolated and characterized [25,26]. For this latter, highly pathogenic fungus, a recombinant AP was generated and shown to induce a protective immune response against a pulmonary infection in mice [26]. However, the role of APs in pathogenesis and immune responses has been most extensively investigated in the opportunistic fungus *C*. *albicans*, providing evidence for previously unsuspected AP “heating” functions. These functions should be considered an important, in some pathologies a likely dominant, addition to the AP broad spectrum of host-impacting, protein degradation activities.

*C*. *albicans* is a eukaryotic pathogen able to thrive equally well as commensal or as pathogen in humans [27]. The fungus can cause lethal systemic infections in immunocompromised or severely debilitated subjects but more frequently cause superficial infections, some of which (for instance, the recurrent vulvovaginal infection [RVVC]) are regularly observed in women without any apparent immune deficit [28]. As detailed below, there is good evidence that the expansion of *C*. *albicans SAP* genes contribute significantly to the virulence program of this fungus in the vaginal disease.

**Fig 2:**
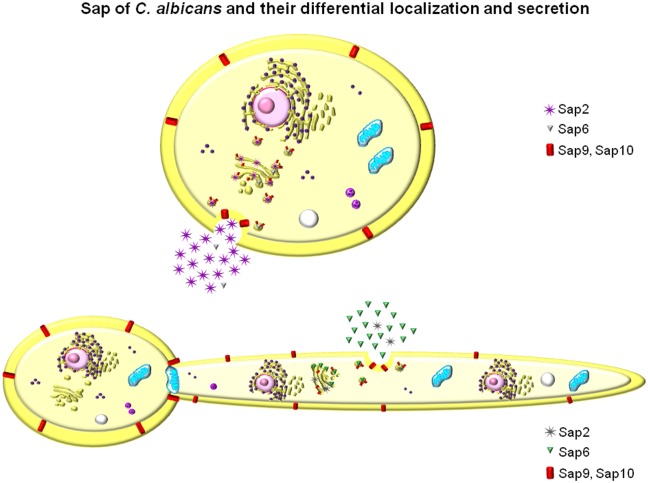
The *SAP* family of *C*. *albicans* contains at least ten proteins with a signal peptide and are secreted, except Sap9 and Sap10, which remain bound to the cell wall. They are characterized by broad spectrum proteolytic ability and virulence properties that are reported to be differentially expressed at different stages and forms of fungus growth and disease. Sap2 (alike Sap1 and Sap3) is active at acidic pH and is dominantly associated with yeast form of growth while Sap6 (alike Sap4 and Sap5) is more active at neutral to slightly alkaline pH Together with the dominant Sap5, Sap6 has been associated with hyphal growth. For details, see [7] and [28].

Of the ten *C*. *albicans* Saps, eight are secreted into the extracellular space (Sap1-8), and two are cell membrane- or cell wall-associated (Sap9-10). They have been reported to play different, although redundant, pathogenic roles, in part associated with the characteristic yeast-to-hypha transition, which is central in the biology and pathogenicity of this fungus [7,29,30] (Fig 2).

## A Blend of Immunoevasion and Immunoactivation

Candidal vaginitis is a disease in which Sap activities seem to have a major impact on host immunity. In fact, in vitro, ex vivo, and animal investigations in distant and immunologically different rodent models (rat and mouse) matched some old clinical data in support of a role for Saps in determining or co-determining the disease [31,32]. However, the mechanisms by which Saps contribute to disease have long remained uncertain, sometimes blurred by the simultaneous expression or activity of other numerous putative virulence traits expressed by this organism [29]. Hypothetical mechanisms mostly focused on Sap capacity to hydrolyze structural proteins of epithelial cells (e.g., E-cadherin) or factors of both innate and adaptive immunity, particularly complement, that allow *C*. *albicans* to prevent or escape from local host immunity and damaging epithelial cells [33–36].

**Fig 3:**
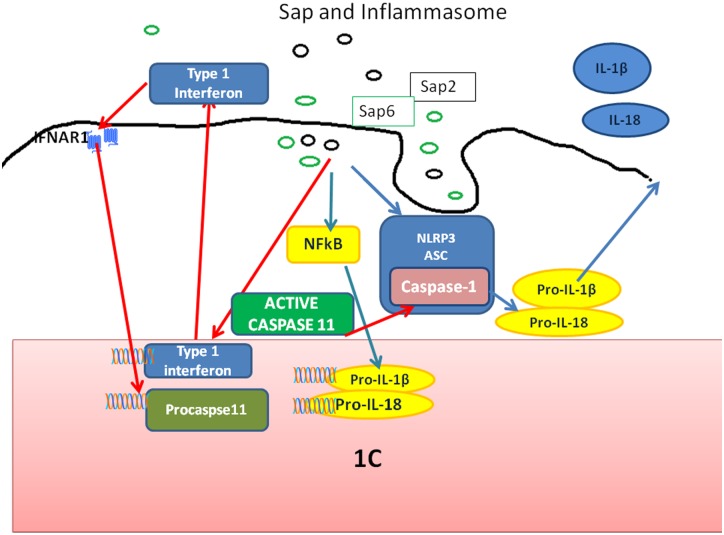
The proposed view of Sap-induced inflammasome activation and inflammasome-dependent cytokine production. Sap2 and Sap6 activate the NLRP3 inflammasome pathway through an early cascade of events, causing upstream NLRP3 inflammasome activation and downstream caspase-1-mediated cytokine production. Late events depend on Sap endocytosis inducing the translocation of NF-κB (p50/p65) into the nucleus, pro-IL-1β and pro-IL-18 synthesis, then (through type I IFN production) caspase-11 activation that cooperates with the NLRP3 inflammasome in triggering downstream caspase-1-mediated cytokine production. For details about this proposed scheme of Sap/inflammasome/caspases activation, see [38], [39], and [42].

More recent data suggest a perhaps more relevant role of Saps in RVVC, i.e., inducing pathogenic inflammation at an inflammation-non-permitted, tolerant body site. It has been demonstrated that some Saps are pro-inflammatory proteins capable of inducing a potent damage response through endocellular inflammasome receptors, particularly the NLRP3 inflammasome, in both hemopoietic and epithelial tissues [37–39]. While adding to the list of other identified or supposed fungal inflammasome activators [40], Saps are the first identified fungal inducers of inflammasome activation of a pure protein nature. Importantly, Sap-induced pro-inflammatory activity, unlike all other Sap functions, does not rely on enzymatic activity [37]. In mouse models of vaginal infection, some Saps (mostly Sap2 and Sap6, or Sap5, depending on the model [33,39]), appear to be responsible for the expression of key immunopathogenic markers of landmark inflammatory events. This includes polymorphonuclear (PMN) cell infiltration and production of pro-inflammatory cytokines, such as IL-1β and IL-18, via the activation of inflammasome-mediated caspase-1 and possibly other caspases [41]. The use of individual or collective subfamily *SAP* knockout strains, human anti-Sap Fragment antigen binding (Fab), and whole transcriptome analyses has shown that inflammation in vaginal disease can be dampened either downstream, by pharmacological inhibition of NLRP3 inflammasome and blockade of IL-1β receptor, or upstream, by specifically affecting Sap production or activity by anti-Sap antibodies or pepstatin A, without affecting the infectious fungus burden [38,39] (Fig 3).

This latter observation suggests that the protective capacity of a recombinant Sap2 vaccine [42] could be mediated by anti-inflammatory antibodies. Interestingly, both NLRP3 and NLRC4 inflammasomes were activated during *C*. *albicans* infection, and NLRC4 and IL-22 were shown to counteract the pathogenic inflammation sustained by NLRP3 [43]. These findings contrast the protective role of NLRP3 inflammasome reported for oral infections [44], further supporting the view that pathogenic and immune mechanisms vary significantly between vaginal and oral candidiasis [45].

## Is *C*. *albicans* SAP-Induced Inflammation Shared by Other AP-Possessing Eukaryotic Pathogens?

Inflammasome activation and inflammatory cytokine cascades are associated with the pathogenesis of a number of diseases caused by other AP-possessing eukaryotic pathogens. Do these APs directly or indirectly participate in inflammation? As an example, do some plasmepsins participate in the inflammation typical of cerebral malaria (CM)? This disease is the worst outcome of infection with *P*. *falciparum*, being lethal or causing severe cognitive deficits in cured patients. Plasmepsin II is actively produced at the disease-critical blood stage of infection. As recently highlighted [46], upon infection with *P*. *falciparum*, the host immune system produces pro-inflammatory cytokines, including IL-1β, which activates endothelial cells that in turn produce CXCL10, a chemo-attractant for mononuclear leukocytes. Very little is known about the specific components of *P*. *falciparum* and other eukaryotic pathogens capable of stimulating the inflammatory cascade. However, plasmepsin 4-deficient *P*. *berghei* do not cause CM in a model of murine malaria [47]. In light of what has been found in *C*. *albicans*, studies on the possible role of other eukaryotic APs in inflammasome activation are worthy of being considered.
